# Response-level processing during visual feature search: Effects of frontoparietal activation and adult age

**DOI:** 10.3758/s13414-019-01823-3

**Published:** 2019-08-02

**Authors:** David J. Madden, Rachel E. Siciliano, Catherine W. Tallman, Zachary A. Monge, Andreas Voss, Jessica R. Cohen

**Affiliations:** 1 Brain Imaging and Analysis Center, Duke University Medical Center, Box 3918, Durham, NC 27710 USA; 2 Department of Psychiatry and Behavioral Sciences, Duke University Medical Center, Durham, NC 27710 USA; 3 Center for Cognitive Neuroscience, Duke University, Durham, NC 27708 USA; 4 Institute of Psychology, University of Heidelberg, Heidelberg, Germany; 5 Department of Psychology and Neuroscience, University of North Carolina at Chapel Hill, Chapel Hill, NC USA

**Keywords:** Attention: neural mechanisms, Attention and aging, Visual search

## Abstract

Previous research suggests that feature search performance is relatively resistant to age-related decline. However, little is known regarding the neural mechanisms underlying the age-related constancy of feature search. In this experiment, we used a diffusion decision model of reaction time (RT), and event-related functional magnetic resonance imaging (fMRI) to investigate age-related differences in response-level processing during visual feature search. Participants were 80 healthy, right-handed, community-dwelling individuals, 19–79 years of age. Analyses of search performance indicated that targets accompanied by response-incompatible distractors were associated with a significant increase in the nondecision-time (*t*0) model parameter, possibly reflecting the additional time required for response execution. Nondecision time increased significantly with increasing age, but no age-related effects were evident in drift rate, cautiousness (boundary separation, *a*), or in the specific effects of response compatibility. Nondecision time was also associated with a pattern of activation and deactivation in frontoparietal regions. The relation of age to nondecision time was indirect, mediated by this pattern of frontoparietal activation and deactivation. Response-compatible and -incompatible trials were associated with specific patterns of activation in the medial and superior parietal cortex, and frontal eye field, but these activation effects did not mediate the relation between age and search performance. These findings suggest that, in the context of a highly efficient feature search task, the age-related influence of frontoparietal activation is operative at a relatively general level, which is common to the task conditions, rather than at the response level specifically.

**Electronic supplementary material:**

The online version of this article (10.3758/s13414-019-01823-3) contains supplementary material, which is available to authorized users.

## Introduction

In visual feature search, the target differs from all of the distractors in some dimension (e.g., color, size, orientation), whereas in conjunction search the target is a combination of distractor features (Treisman, 1988, 2004). As a result, feature search performance is highly efficient, as defined by minimal differences in reaction time (RT) as function of the number of items in the search display (Wolfe, 2014). This increased efficiency may reflect various mechanisms, including an early-stage parallel search of the display, as originally proposed by Treisman (1980), but also the degree of similarity between the target and distractors (Duncan & Humphreys, 1989; Treisman, 1991) and the influence of top-down attention (Müller, Heller, & Ziegler, 1995; Wolfe, Butcher, Lee, & Hyle, 2003).

An intriguing property of visual feature search is that it is relatively resistant to age-related decline. During normal human aging, performance of conjunction search tasks becomes less efficient, as reflected in increased RT-display size slopes and increased error rates (Hommel, Li, & Li, 2004; Humphrey & Kramer, 1997; Madden & Whiting, 2004; Rabbitt, 2017), consistent with the increase in RT that is typically observed for older adults as a function of increasing task demands (Salthouse, 1985, 1996). Feature search performance is affected minimally by aging, other than an overall age-related increase in RT. Feature-search RT-display size slopes typically remain near zero for both older and younger adults (Plude & Doussard-Roosevelt, 1989; Whiting, Madden, Pierce, & Allen, 2005). The age-related decline in conjunction search appears to reflect one aspect of a more general decline in attentional and executive functioning, as well as general slowing, although some forms of top-down attentional control and spatial focusing of attention remain relatively preserved with aging (Kramer & Madden, 2008; Madden, 2007; McAvinue et al., 2012).

Neuroimaging studies of younger adults have established that attention-demanding tasks engage a widely distributed frontoparietal network, including the frontal eye field (FEF), inferior parietal lobule (IPL), and superior parietal lobule (SPL), which can extend to include visual processing and motor response-related regions, such as lateral and inferior prefrontal cortex, anterior cingulate, supplementary motor and premotor regions (Corbetta, Patel, & Shulman, 2008; Katsuki & Constantinidis, 2014; Nobre & Mesulam, 2014; Wei, Muller, Pollmann, & Zhou, 2011). The overall topography of the frontoparietal networks related to attention and response processing appears to hold across adult age (Madden & Monge, 2019; Madden et al., 2007; Müller-Oehring, Schulte, Rohlfing, Pfefferbaum, & Sullivan, 2013). Older adults have, in some instances, however, exhibited increased task-related activation, particularly in dorsolateral prefrontal regions, which may be a compensatory mechanism to support task performance (Davis, Dennis, Daselaar, Fleck, & Cabeza, 2008; Dennis & Cabeza, 2008; Spreng, Wojtowicz, & Grady, 2010). In the context of conjunction search, aging is associated with an enhancement of functional connectivity between regions (Geerligs, Saliasi, Maurits, Renken, & Lorist, 2014), and enhancement of the relation between neural activation and performance (Madden, Parks, Tallman, Boylan, Hoagey, Cocjin, Johnson, et al., 2017a; Monge et al., 2017).

Little is known, however, regarding the neural mechanisms of visual feature search in relation to age. Is the relative preservation of visual feature search performance with adult age due to a constancy in the underlying neural functioning, or does an age-related difference in task-related activation support the age similarity in the behavioral measure of search performance? In a study of distraction during feature search, Madden et al. (2014) reported that although visually salient distractors (color singletons) were more disruptive to target detection for older adults relative to younger adults, and that activation within the frontoparietal network was related to distraction, age was related independently to activation and to performance. This pattern is consistent with an age constancy of search-related neural functioning.

In this experiment, we used event-related fMRI to investigate the relation of age to neural activation for response-level processing during visual feature search. The nontarget (distractor) items within each display were response-compatible, response-incompatible, or neutral in relation to the target. This allowed the distinction between the facilitatory effects of response-compatible distractors from the inhibitory effects of response-incompatible distractors. Behavioral studies of younger adults have found that response-incompatible distractors during visual search lead to slower and less accurate target identification, even when search is highly efficient (Mortier, Theeuwes, & Starreveld, 2005; Starreveld, Theeuwes, & Mortier, 2004). Similarly, behavioral studies suggest that the selection and control of task-relevant responses is a specific locus of age-related decline (Augustinova, Clarys, Spatola, & Ferrand, 2018; Hartley, 2001; Machado, Devine, & Wyatt, 2009; Maylor & Lavie, 1998; Nebes, 1978; Proctor, Vu, & Pick, 2005; Spieler, Balota, & Faust, 1996). In neuroimaging studies, response-level processing has been associated with age-related compensatory recruitment of neural activation (Langenecker, Nielson, & Rao, 2004; Milham et al., 2002; Nielson, Langenecker, & Garavan, 2002; Paxton, Barch, Racine, & Braver, 2008; Sebastian et al., 2013), but these previous studies have used either prepotent responses (e.g., Stroop) or relatively complex target-response mapping. Age constancy in the magnitude of the behavioral effects for response competition and inhibition has also been reported (Atwi et al., 2018; Hsieh & Lin, 2014; Kramer, Humphrey, Larish, Logan, & Strayer, 1994; Madden & Langley, 2003), and thus the specific attentional demands leading to the age-related differences are not yet clear. To our knowledge, no previous study of age-related differences in response-level processing during visual feature search has been reported.

To more accurately characterize the effects of response-level processing, beyond mean RT and error rate, we analyzed search performance with the diffusion decision model (Ratcliff, 1978; Ratcliff, Smith, Brown, & McKoon, 2016; Voss, Nagler, & Lerche, 2013a; Voss, Voss, & Lerche, 2015). This analytic technique uses the full distributions of RT and error rate to estimate parameters for the decisional and nondecisional aspects of two-choice discrimination responses, as well as the overall conservativeness of the evidence threshold (i.e., cautiousness). Analyses using the diffusion model have consistently documented an age-related increase in the nondecision time parameter (*t*0) that represents the time required for visual encoding and motor response initiation (Ratcliff, 2008; Ratcliff, Thapar, Gomez, & McKoon, 2004). An age-related decline in the rate of evidence accumulation in decisional processes (drift rate, *v*) has also been reported, though it is more task-dependent (Madden et al., 2009; Spaniol, Madden, & Voss, 2006; Yang, Bender, & Raz, 2015). Cautiousness, as reflected in the boundary separation parameter (*a*), also tends to increase with age (Ratcliff, 2008), though again with exceptions (Monge et al., 2017). Neuroimaging studies have found that age-related slowing of drift rate is associated with task-related frontoparietal activation and connectivity (Madden et al., 2010; Monge et al., 2017), but these previous studies did not isolate response-level processes.

In this investigation of age-related differences in neural activation and visual feature search, we focused on two issues. First, at the behavioral level, what are the age-related differences in response-level processing? Assuming that the decisional processes modeled by drift rate should be minimal for feature search, we hypothesized that age-related differences should be expressed more prominently in nondecision time than in drift rate (Ratcliff, 2008; Ratcliff et al., 2004), in view of the age-related constancy noted for feature search RT slopes (Plude & Doussard-Roosevelt, 1989; Whiting, Madden, Pierce, & Allen, 2005). We expected that the increase in nondecision time associated with response-incompatible items, in particular, would become more pronounced with increasing age (Hartley, 2001; Proctor et al., 2005). Second, does age have independent relations to search-dependent activation and behavioral performance, or does an age-related difference in activation support search performance? Madden et al. (2014) reported that, in feature search, age had independent effects on task-related activation and on the slowing associated with the presence of a salient distractor. We hypothesized that if response-level processing is a more prominent locus of age-related decline, then age should not have independent effects on activation and performance. Instead, task-related activation should be a mediator of the age-search performance relation, such that the effect of age on performance is reduced significantly when controlled for activation (Hayes, 2013; Hayes & Rockwood, 2017).

This type of mediating influence could occur in various patterns. For example, even if search performance is constant with age, increased activation could be operating in a manner to support that constancy. Alternatively, if response-incompatible distractors lead to a slowing of search performance that is relatively greater for older adults, increasing task-related activation may reflect the neural resources needed for older adults to control the competing responses. In either case, age should have an indirect effect on feature search performance, operating through regional activation, and mediation should be specific to the effect of response-incompatibility rather than a general property of all the task conditions.

## Materials and methods

### Participants

The research was conducted in accordance with the Code of Ethics of the World Medical Association (Declaration of Helsinki) for experiments involving humans. Participants gave written informed consent for a protocol approved by the Duke University Medical Center Institutional Review Board. The participants were 80 healthy, community-dwelling individuals (43 women) 19–79 years of age. All participants reported that they were right-handed, had completed at least 12 years of education, were free of significant health problems (including atherosclerosis, neurological and psychiatric disorders), and were not taking medications known to affect cognitive function or cerebral blood flow (except antihypertensive agents).

Participants completed an initial screening session and then the MRI testing approximately 1 month later. The screening session included an abbreviated version of the visual search task to be performed during scanning, as well as visual sensory and psychometric tests (Table 1). The final sample of 80 participants comprised 26 individuals between 19 and 39 years of age, 25 individuals between 40 and 59 years of age, and 29 individuals between 60 and 79 years of age.

**Table 1:** Participant Characteristics

*M*		age *r*	
Education (years)	16.963	(1.977)	0.450***
MMSE	29.100	(1.014)	-0.241*
Vocabulary	55.213	(6.332)	0.148*
Digit Symbol RT	1621.000	(323.649)	0.650***
Color Vision	13.913	(0.363)	-0.243*
Visual Acuity	-0.081	(0.097)	0.341**

*Note*. *n* = 80. *M* = mean, with *SD* in parentheses; age *r* = correlation with age; MMSE = raw score on Mini-Mental State Exam (Folstein et al., 1975); Vocabulary = raw score on the Wechsler Adult Intelligence Scale III (Wechsler, 1997); Digit Symbol RT = mean reaction time on a computer test of digit-symbol coding (Salthouse, 1992); Color Vision = score on Dvorine color plates (Dvorine, 1963); Visual Acuity = logarithm of the minimum angle of resolution (MAR), for the Freiburg Visual Acuity Test (FRACT; Bach, 1996). Log MAR of 0 corresponds to Snellen 20/20, with negative values corresponding to better resolution. Thus, the positive correlation for acuity represents age-related decline in this measure.

*p < .05

**p < .01

***p < .001

### Visual search task

**Fig. 1:**
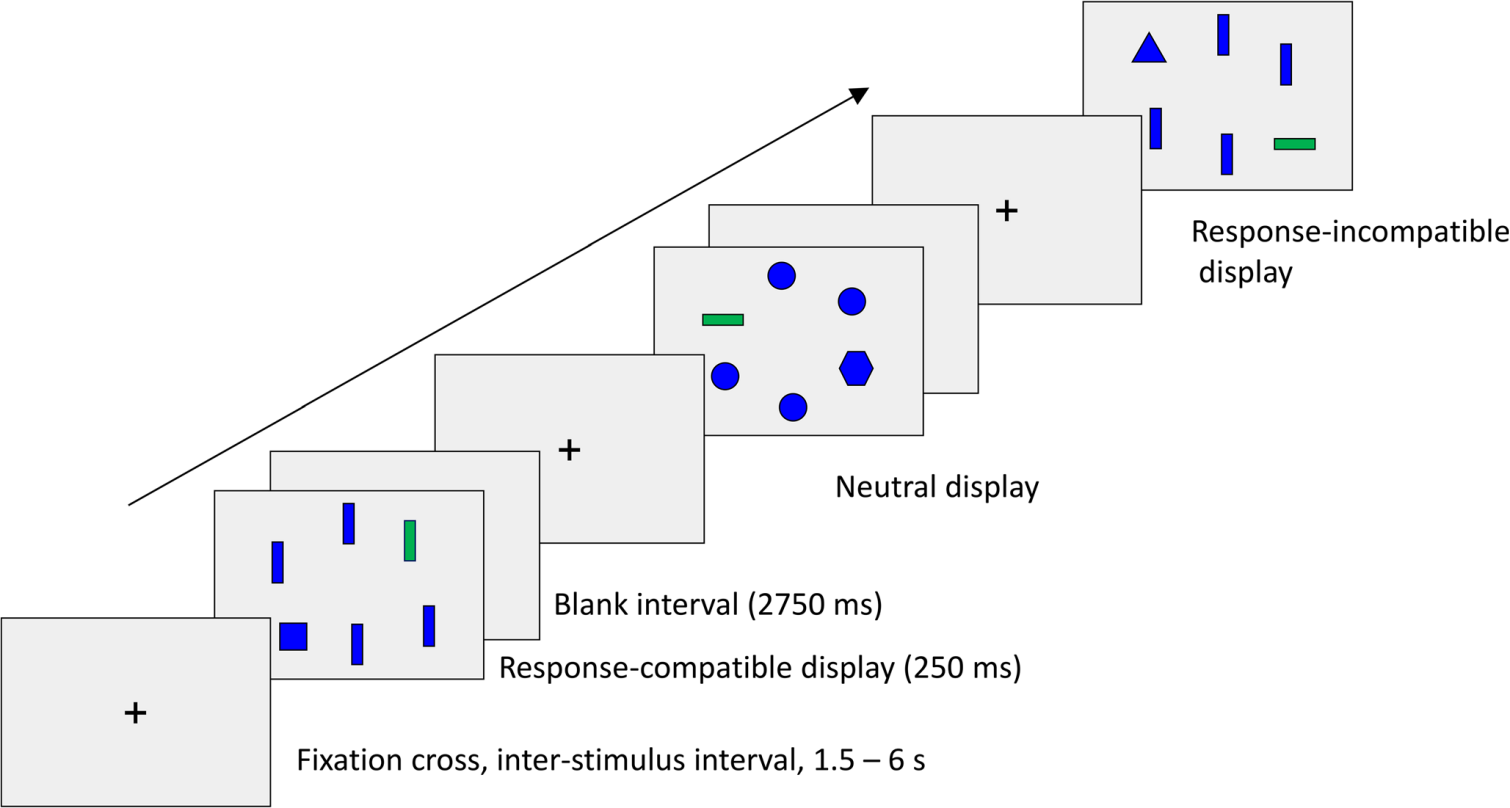
Task structure. A single display appeared on each trial. Each display contained a color singleton bar and a shape singleton. The participant’s task was to respond about whether the color singleton was either horizontal or vertical. The other four display items were shapes that were compatible, neutral, or incompatible with the correct response. The inter-stimulus interval was jittered between 1.5 – 6 s

While in the scanner, participants performed a form of feature search in which the task was to make a vertical/horizontal response regarding the target, a color singleton bar. As illustrated in Fig. 1, each display comprised six items, the target bar and five nontarget (distractor) shapes. All distractors were the same color (e.g., a blue target bar among green distractors, or vice versa). For response-compatible trials, four of the distractors were bars with the same orientation as the target. For response-incompatible trials, four of the distractors were bars in the opposite orientation to the target. Neutral displays contained four instances of a shape other than horizontal or vertical bars (ovals, triangles, or rectangles). To emphasize color as the relevant feature for defining the target, we included, as the fifth distractor within each display, a shape that was different from both the target and the other distractors (but shared the distractor color). This item was always located diagonally across from the target. Thus, the target was always a color singleton but was not additionally a shape singleton. Across trials within each scanner run, the displays varied pseudo-randomly among the three task conditions of response-compatible, response-incompatible, and neutral displays.

Displays were presented using E-Prime 2.0 (Psychology Software Tools, Sharpsburg, PA, USA). The value of color defining the target (blue or green) was constant for each participant and counterbalanced across participants. The items in the display were isoluminant and presented on a black background. The bars were approximately 1.67° x 0.40°, and the other shapes (ovals, triangles, and rectangles) were similar in size. The display items were in an approximately circular (10° diameter) arrangement; the center of each display location was jittered slightly (30–38 pixels) across trials, to reduce adaptation effects. The target bar could appear at one of four locations, at approximately 2 o’clock, 4 o’clock, 8 o’clock, and 10 o’clock, within the circular display. Participants first completed one practice run of 20 trials (during structural imaging), followed by four functional imaging runs of 48 trials each, for a total of 192 trials. The 48 trials within each fMRI scanning run were a randomized sequence with two trials for each combination of response compatibility, target location, and target orientation. Thus, across the 192 test trials, there were 64 trials for each of the three compatibility conditions, with the target occurring at each of the four possible target locations 16 times (with eight vertical and eight horizontal instances).

At the presentation of each display, participants indicated their vertical/horizontal decision regarding the color singleton target via a button-press response, using their right index and middle fingers and two buttons on a hand-held, fiberoptic response box (Current Designs, Philadelphia, PA, USA). Participants were instructed to respond as quickly as possible without sacrificing accuracy, and the assignment of the horizontal/vertical response to the response buttons was balanced across participants. Each trial began with a white fixation cross with variable duration (jitter), followed by the six-item display for a duration of 250 ms, then a 2,750-ms response period, during which the display was black. We measured RT from display onset. Following the response period, the fixation cross returned to begin the next trial. The jitter duration was varied among values of 1,500, 3,000, 4,500, and 6,000 ms (average jitter = 2,313 ms) defined by multiples of the fMRI repetition time (TR) value (1,500 ms). The jitter values and trial order across conditions were randomized and optimized using the Optseq2 program (Dale, 1999; http://surfer.mnr.mgh.harvard.edu/optseq). No feedback regarding response accuracy was provided.

### Diffusion decision model parameters for reaction time

**Fig. 2:**
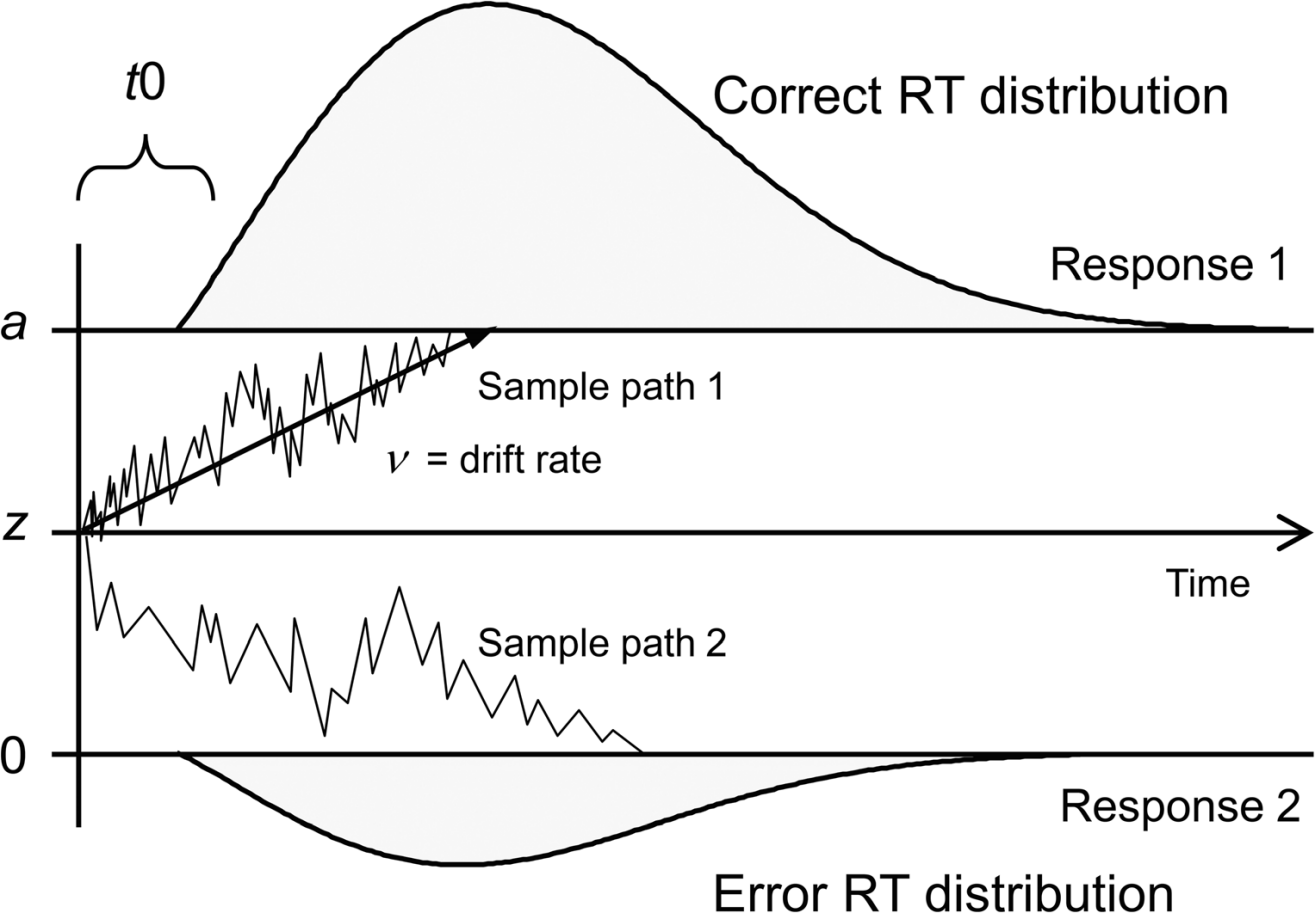
Diffusion decision model of choice reaction time (Ratcliff et al., 2016; Voss et al., 2015). Each response is modeled as the combined effects of several processes represented by individual parameters. The drift rate, *v*, is the rate of evidence accumulation towards one of two response alternatives, one of which is a correct response. The boundary separation, *a*, represents the amount of evidence required for a response, with higher *a* values reflecting increased cautiousness. Nondecisional processes, comprising primarily motor response and visual sensory encoding of the display, contribute to a separate parameter, *t*0

We fit the diffusion decision model (Fig. 2) to individual data sets using the fast-dm program (Voss & Voss, 2007; Voss et al., 2015). Monte Carlo simulation indicated a good fit of the model to the data, and additional analyses of RT for correct and incorrect responses did not reveal significant differences in the speed/accuracy emphasis as a function of task condition or age (Supplementary Material and Table S1).

Our primary analyses were regression and repeated-measures analysis of variance (ANOVA) on the drift rate (*v*), nondecision time (*t*0), and boundary separation variables. Power analyses (Faul, Erdfelder, Lang, & Buchner, 2007) indicated that, with 80 participants, a Pearson correlation *r* value of 0.35 (*r*^2^ = 0.123) would be detected as significant, at alpha *p* = 0.05 (two-tailed), with a power of 0.90. For ANOVA, the difference between two within-subjects’ conditions, corresponding to a small-to-medium effect size *f* (Cohen, 1988) of 0.176 (partial eta-squared, η_*p*_^2^, of 0.03), would be detected at alpha *p* = 0.05 (two-tailed) with a power of 0.87. The η_*p*_^2^ values are reported for ANOVA effects.

### MRI data acquisition

We collected imaging data on a 3.0 T GE MR750 whole-body MRI scanner (GE Healthcare, Waukesha, WI, USA) equipped with a 60-cm bore, 50 mT/m gradients, and a 200 T/m/s slew rate. An eight-channel head coil was used for radio frequency (RF) reception. Participants wore earplugs to attenuate scanner noise and foam pads surrounded the head to reduce head motion. We first acquired three-plane (straight axial/coronal/sagittal) localizer fast spin echo (FSE) images that defined a volume for data collection, using a semi-automated high-order shimming program to ensure global field homogeneity. Following these were four event-related T2*-weighted (functional) runs imaging sensitive to the blood oxygen-level dependent (BOLD) signal. Participants performed the search task during these runs. The event-related runs were followed by one run of T1-weighted anatomical imaging, for registration with the functional images. The protocol also included several data sets not reported here: two diffusion-weighted imaging (DWI) runs, two resting-state functional imaging runs, and one T2-weighted fluid attenuated inversion recovery (FLAIR) imaging run.

For the T1-weighted anatomical images, 166 straight axial slices were acquired with a 3D fast inverse-recovery-prepared spoiled gradient recalled (SPGR) sequence, with TR = 8.10 ms, echo time (TE) = 3.18 ms, inversion recovery time (TI) = 450 ms, field of view (FOV) = 256 mm, flip angle = 12°, voxel size = 1 x 1 x 1 mm, 256 x 256 matrix, and a sensitivity encoding (SENSE) factor of 2, using the array spatial sensitivity encoding technique and extended dynamic range.

Event-related functional imaging comprised 29 contiguous slices acquired at an axial oblique orientation, parallel to the AC-PC plane; TR = 1,500 ms, TE = 27 ms, FOV = 240 mm, flip angle = 77°, voxel size = 3.75 x 3.75 x 4 mm, 64 x 64 matrix, and a SENSE factor of 1. For each event-related run, 188 brain volumes were collected, the first four volumes of which were discarded to allow scanner equilibrium.

### fMRI preprocessing and modeling

We assessed data quality using an in-house tool that quantifies several metrics including signal-to-noise ratio (SNR), signal-fluctuation-to-noise ratio (SFNR), participant motion, and measures of voxel-wise standard deviation (Friedman & Glover, 2006; Glover et al., 2012). Data were also inspected visually for artifacts and blurring. Preprocessing steps including motion correction and high-pass temporal filtering (cut off = 90.0 s) were conducted in FSL 5.0.1 (Smith et al., 2004; http://www.fmrib.ox.ac.uk/fsl) and FEAT version 6.0. Structural brain images were skull-stripped using the FSL brain extraction tool (Smith, 2002). Functional images were then corrected for slice-timing and head motion using six rigid-body transformations using FSL MCFLIRT (Jenkinson, Bannister, Brady, & Smith, 2002). These transformations were included as nuisance covariates in the FSL model. Using a motion threshold of 2.5 mm in any direction within a run, < 0.15% of the total volumes were modeled as motion-influenced. Functional images of each participant were coregistered to structural images in native space and then normalized to the MNI152 T1 template (Montreal Neurological Institute, Montreal, Canada) using a combination of affine and non-linear registrations (Greve & Fischl, 2009; Jenkinson et al., 2002; Jenkinson & Smith, 2001). The functional images were then spatially smoothed with a 5-mm Gaussian kernel.

Voxelwise analyses were conducted within FSL, with a double γ function modeling the hemodynamic response on each trial for each participant. The modeling included explanatory variables for the correct-response trials for each of the three task conditions (compatible, incompatible, and neutral trials) and a fourth variable representing errors (incorrect or omitted responses). The model also included several nuisance regressors: each independent event’s temporal derivative, six motion regressors, and two regressors representing the white matter and cerebral spinal fluid (CSF) time-series. The white matter and CSF time-series were obtained by segmenting each participant’s T1 image, binarizing the white matter and CSF segments (threshold = 99% probability), resampling the segments to functional space, and extracting the averaged white matter and CSF time series.

Contrasts were defined within-run for each participant using the three explanatory variables for the task conditions (compatible, incompatible, and neutral). Each trial type was compared to the implicit baseline. As a measure of overall task-related activation, the modeled hemodynamic response for the correct-response trials was averaged across the three task conditions. Differences between trial types (each relative to the implicit baseline) were contrasted to identify specific effects of response-compatible and -incompatible trials. The modeled data were then averaged across the four experimental runs for each participant and analyzed with one-sample t-tests using FMRIB Local Analysis of Mixed Effects (FLAME 1 & 2) (Beckmann, Jenkinson, & Smith, 2003; Woolrich, Behrens, Beckmann, Jenkinson, & Smith, 2004). The contrast for overall task-related activation includes all search-related encoding, decision, and response effects, and thus the cluster threshold for this contrast was set at *z* > 5.0, GRF-corrected at *p* < .0001. The contrasts between the individual task conditions were cluster thresholded at *z* > 2.3, GRF-corrected at *p* < .05.

Drift rate and nondecision time were analyzed as behavioral covariates (demeaned continuous variables), in separate models, for overall activation (all trials > baseline) and task condition contrasts. The model with boundary separation as a covariate was limited to the overall activation model, as boundary separation is assumed not to vary at the level of individual trials (Ratcliff et al., 2016; Voss et al., 2015). To identify regions of age-related activation that may have occurred outside of those identified in the overall and task condition contrasts, we also conducted analyses of all of the contrasts with age as a covariate.

The local maxima of significant clusters were identified and labeled using the Harvard-Oxford Atlas within FSL, the Duvernoy atlas (Duvernoy, 1999), and the Nonlinear Yale MNI to Talairach Conversion Algorithm (Lacadie, Fulbright, Rajeevan, Constable, & Papademetris, 2008; http://sprout022.sprout.yale.edu/mni2tal/mni2tal.html). Coordinates are reported in MNI space, and results in figures are overlaid on the MNI template brain in radiological convention (left = right). Additional analyses were conducted by using the coordinates of these local maxima as the centers of 8-mm-diameter spherical regions of interest (ROIs). For each participant, we used FSL Featquery to estimate the percentage signal change for each ROI, averaged across all voxels within the ROI. The ROI data were analyzed using SAS 9.4 (SAS Institute, Inc., Cary, NC, USA) within the general linear model. For task contrasts with several local maxima, we estimated a general factor of activation for the corresponding ROIs. Following a procedure similar to that of Salthouse et al. (2015) and Madden, Parks, Tallman, Boylan, Hoagey, Cocjin, et al. (2017b), we used the unrotated first factor, from a principal axis factor analysis of the data for the relevant ROIs, as an estimate of a general factor of activation.

To determine whether the activation effects were significant mediators of the relation between age and search performance, mediation analyses were conducted with the PROCESS macro for SAS (Hayes, 2013), with age as a predictor, search performance (drift rate, nondecision time, and boundary separation) as the outcome variable, and activation from the ROIs as potential mediators. Parameter estimates and 95% bootstrap confidence intervals were based on 10,000 bootstrap samples. Significant indirect effects were defined by a confidence interval not including zero.

## Results

### Search performance

**Fig. 3:**
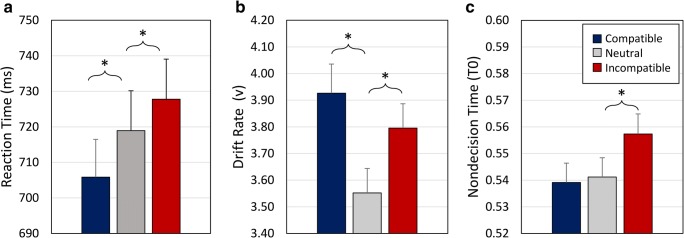
Panel A = mean reaction time (RT). Panel B = drift rate (*v*). Panel C = nondecision time (*t*0)

Averaged across age, accuracy was 97% within each of the three task conditions. Mean values for correct-response RT, drift rate, and nondecision time are presented Fig. 3. An ANOVA of mean RT with task condition as a within-subjects variable yielded a significant main effect of task condition *F*(2, 79) = 28.53, *p* < 0.0001, η_*p*_^2^ = 0.419. The 9-ms increase in RT for incompatible trials relative to neutral trials was significant, *F*(1, 79) = 10.33, *p* < 0.0019, η_*p*_^2^ = 0.116, as was the 13-ms decrease in RT for compatible trials relative to neutral trials, *F*(1, 79) = 24.62, *p* < 0.0001, η_*p*_^2^ = 0.238 (Fig. 3, Panel A). Difference scores representing the RT benefit for the compatible trials and the RT cost for the incompatible trials, relative to neutral trials, were not correlated significantly with age, with |r| < 0.14 in each case.

The ANOVA of drift rate yielded a significant effect of task condition, *F*(2, 79) = 8.51, *p* < 0.0003, η_*p*_^2^ = 0.097. The presence of a response-relevant distractor, regardless of its compatibility with the target, improved the decision process. Relative to the neutral trials, drift rate was higher for both compatible trials, *F*(1, 79) = 16.28, *p* < 0.0001, η_*p*_^2^ = 0.171, and incompatible trials, *F*(1, 79) = 8.36, *p* < 0.005, η_*p*_^2^ = 0.096 (Fig. 3, Panel B).

**Fig. 4:**
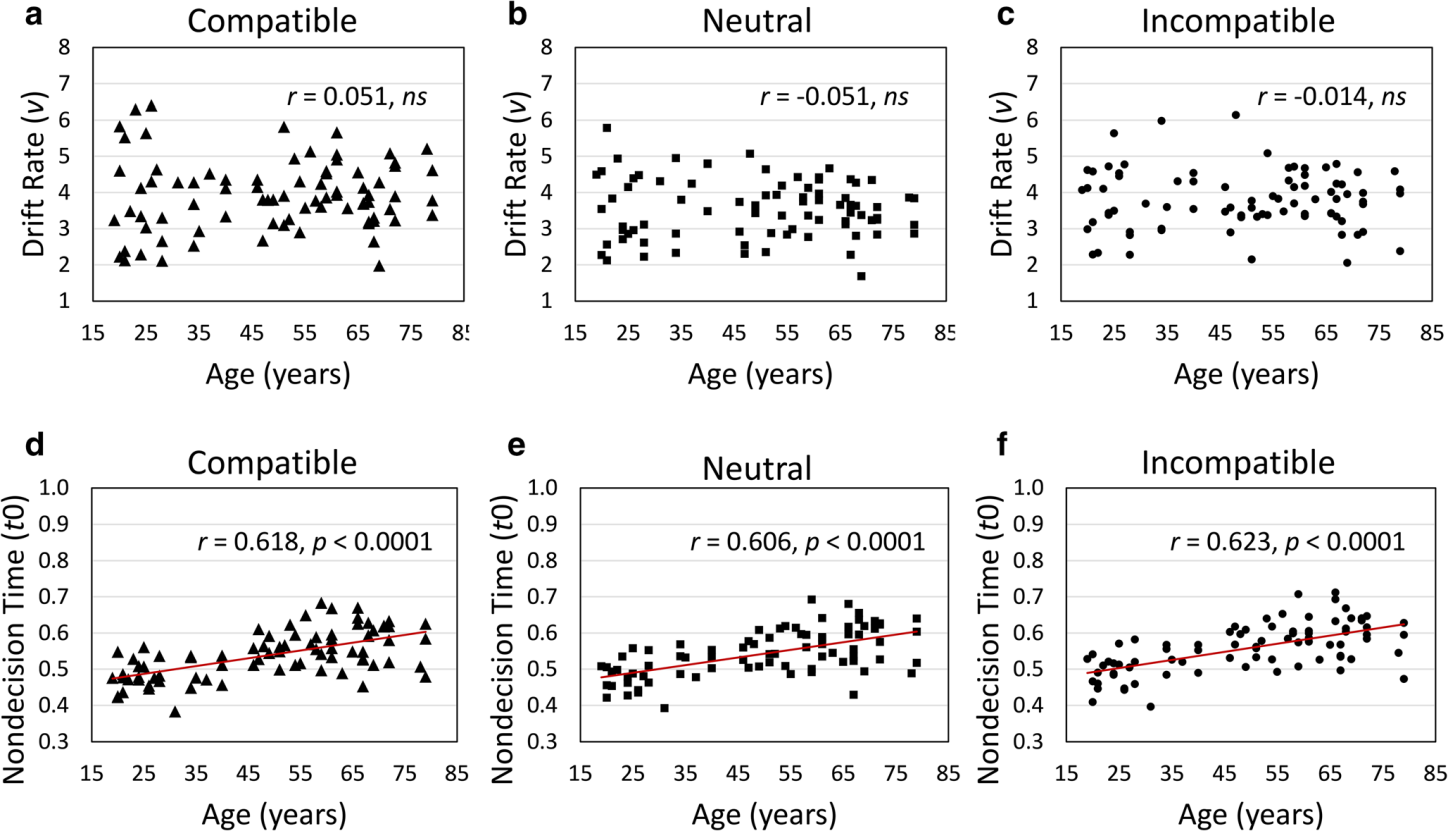
Panels A–C = drift rate as a function of age; with Panel A = compatible condition, Panel B = neutral condition, and Panel C = incompatible condition. Panels D–F = nondecision time as a function of age; with Panel D = compatible condition, Panel E = neutral condition, and Panel F = incompatible condition

Drift rate values are presented as a function of age and task condition in Fig. 4 (Panels A–C). Within each condition, the correlation between age and drift rate was not significant, with |*r*| < 0.06, in each case. Difference scores representing the drift rate benefits for the compatible and incompatible trials were not correlated with age, with |*r*| < 0.12, in each case.

The nondecision time values also varied significantly across the task conditions, *F*(2, 79) = 24.55, *p* < 0.0001, η_*p*_^2^ = 0.237, as a result of a selective slowing of nondecisional processes by response-incompatible distractors. Mean nondecision time was higher for incompatible trials relative to neutral trials, *F*(1, 79) = 26.81, *p* < 0.0001, η_*p*_^2^ = 0.253, whereas the nondecision time values for neutral and compatible trials did not differ, *F*(1, 79) = 0.57, *ns*, η_*p*_^2^ = 0.007 (Fig. 3, Panel C).

Nondecision time values are presented as a function of age and task condition in Fig. 4 (Panels D–F). Within each condition, nondecision time increased significantly with age, with *r* > 0.60, *p* < 0.0001, in each case. Comparison of the correlations across the conditions with Steiger’s Z, however, did not detect any difference in the magnitude of the age-related effect between task conditions. The regression equations for the age-related effects in nondecision time indicated that the increase in nondecision time with age was ~2 ms per year across the three task conditions. Difference scores for compatible and incompatible nondecision time, relative to neutral nondecision time, were not correlated with age, with |*r*| < 0.10, in each case.

The mean value for boundary separation was 1.24 (*SD* = 0.273) and did not vary significantly with age (*r* = 0.145, *p* < 0.20).

### fMRI analyses

**Fig. 5:**
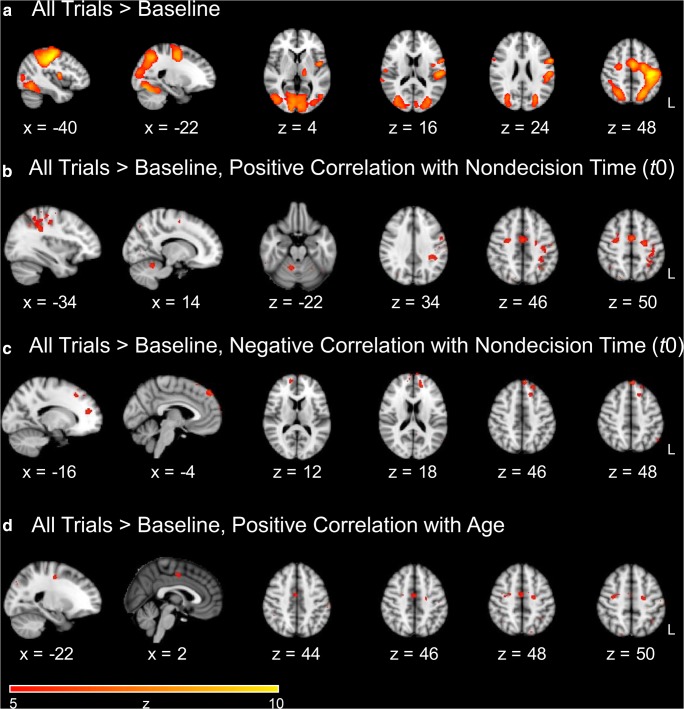
Voxelwise activation for all trials relative to the implicit baseline. Panel A = mean activation. Panel B = positive correlation with nondecision time. Panel C = negative correlation with nondecision time. Panel D = positive correlation with age. Activation is thresholded at *Z* = 5.0, *p* < 0.0001, Gaussian random field (GRF) corrected at *p* < 0.05. Activation is overlaid on the Montreal Neurological Institute (MNI) template brain in radiological convention (left = right)

*Overall task-related activation.* The all trials > baseline contrast (Table 2 and Fig. 5, Panel A) yielded six clusters centered in dorsal frontoparietal cortex, but also including occipital and subcortical (thalamus) regions. The most extensive cluster included local maxima in the left precentral and postcentral gyri. For this contrast, no clusters covaried significantly with either drift rate or boundary separation. Six clusters, however, including FEF bilaterally and left inferior parietal lobule (IPL), covaried positively with nondecision time (Table 2 and Fig. 5, Panel B). Four clusters, primarily in left medial and superior frontal gyri, covaried negatively with nondecision time (Table 2 and Fig. 5, Panel C). With age as the covariate for this contrast, three clusters, in the FEF bilaterally, covaried positively (Table 2 and Fig. 5, Panel D). These clusters were a subset of those exhibiting the positive correlation with nondecision time. No clusters for this contrast were correlated negatively with age.

**Table 2:** Activation for All Trials Relative to Baseline, and with Diffusion Decision Model Parameters and Age as Covariates

Cluster	Max Z	Size (voxels)	Hem	MNI Coord (mm)			BA
				*x*	*y*	*z*	
**All Trials > Baseline**							
Postcentral gyrus	10.30	25925	L	-38	-24	48	1
Precentral gyrus	8.66	921	L	-58	6	24	6
Thalamus	6.72	162	L	-14	-22	2	50
Central opercular cortex	6.49	45	R	60	-16	16	40
Supramarginal/Sup temp gyrus	5.87	31	R	68	-36	16	22
Precentral gyrus	5.78	88	R	60	8	28	6
**All Trials > Baseline, Positive Correlation with Drift Rate (*v*)**							
(no significant clusters)							
**All Trials > Baseline, Negative Correlation with Drift Rate (*v*)**							
(no significant clusters)							
**All Trials > Baseline, Positive Correlation with Nondecision Time (*t*0)**							
Inferior parietal lobule	6.09	427	L	-34	-38	34	40
FEF/Precentral gyrus	6.60	303	L	-22	-14	50	6
FEF/Precentral gyrus	6.03	182	R	26	-6	46	6
Anterior Cingulate	6.79	178	C	0	-4	50	24
Cerebellum	6.02	85	R	14	-52	-22	
Precentral gyrus	5.57	47	L	-52	0	30	6
**All Trials > Baseline, Negative Correlation with Nondecision Time (*t*0)**							
Superior frontal gyrus	6.11	102	L	-16	46	18	10
Medial frontal gyrus	6.85	68	L	-4	50	46	9
Superior frontal gyrus	5.81	58	L	-20	38	46	8
Superior frontal gyrus	5.93	47	R	14	58	12	10
**All Trials > Baseline, Positive Correlation with Boundary Separation (*a*)**							
(no significant clusters)							
**All Trials > Baseline, Negative Correlation with Boundary Separation (*a*)**							
(no significant clusters)							
**All Trials > Baseline, Positive Correlation with Age**							
FEF/Precentral gyrus	5.87	39	L	-22	-14	48	6
FEF/Precentral gyrus	5.72	40	R	32	-10	50	6
ACC/Supplementary motor cortex	5.56	56	R	2	-10	48	24
**All Trials > Baseline, Negative Correlation with Age**							
(no significant clusters)							

*Note.* All Trials > Baseline represents activation for all trials relative to jittered inter-trial interval. Max Z = highest Z values within each cluster; Hem = hemisphere; R = right; L = left; C = center; MNI = Montreal Neurological Institute; Coord = coordinates; Sup Temp = Superior

Temporal; FEF = frontal eye field; BA = Brodmann area. Cluster regions are labeled from the Harvard-Oxford Atlas as implemented within FSL (Smith et al., 2004; http://www.fmrib.ox.ac.uk/fsl). Activation for the All Trials > Baseline contrasts are thresholded at Z = 5.0, *p* < 0.0001

**Fig. 6:**
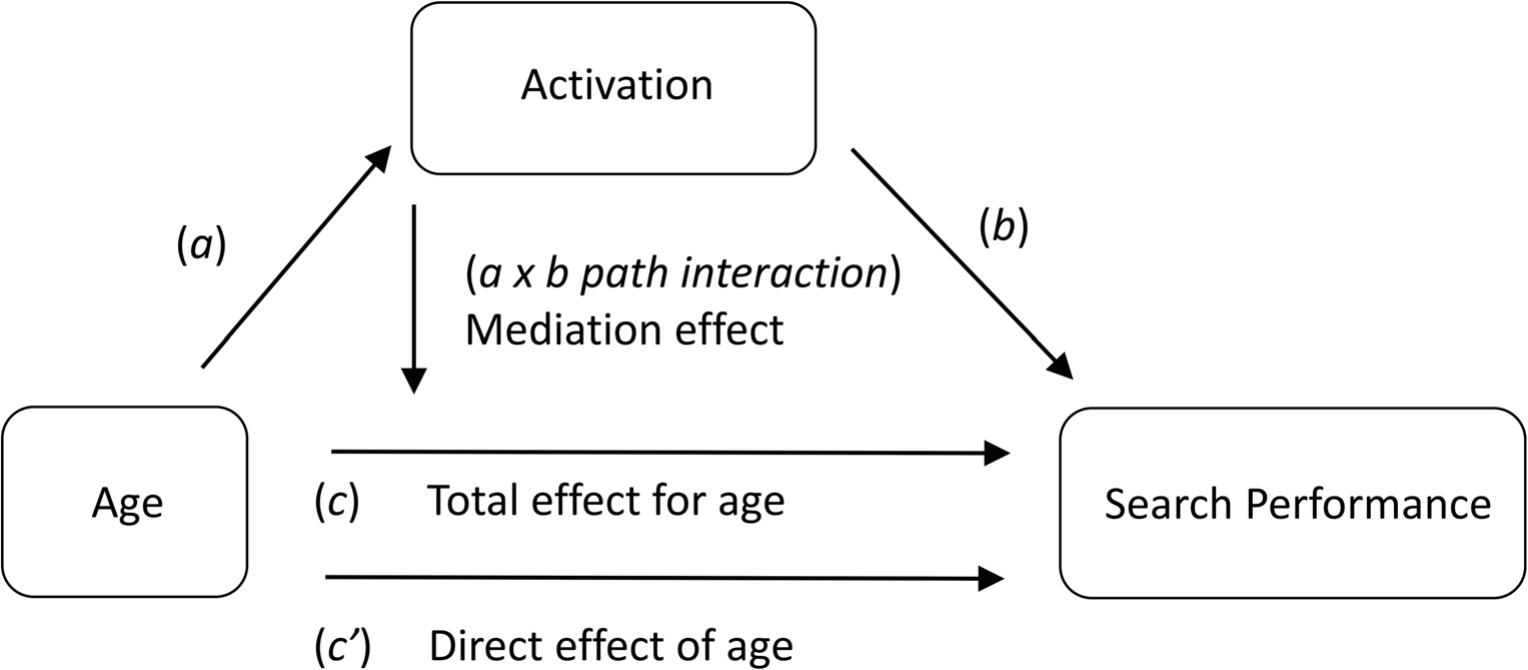
Mediational model

*Positive covariation between overall activation and nondecision time.* With the method described previously (*fMRI preprocessing and modeling* section), we estimated a general factor of activation for the six frontoparietal ROIs covarying positively with nondecision time. The squared multiple correlation of the six ROIs with this factor was 0.862. We conducted a mediation analysis (Hayes, 2013), with the general activation factor as a mediator of the relation between age (as the predictor) and nondecision time (as the outcome). As illustrated in Fig. 6, in this model, the *a* path represents the relation between age and the mediator, the *b* path represents the relation between the mediator and the outcome variable, covaried for age, and the *c* path represents the total effect of age on the outcome variable. The mediating effect of the activation is the *a* x *b* path interaction, which can be expressed alternatively as the degree to which the direct effect of age (*c’* path), is reduced relative to the total effect (*c* path), by including the mediation effect. This analysis demonstrated that the general activation factor was a significant mediator of the age-nondecision time relation (Table 3, Model 1). However, the direct effect of age on nondecision time remained significant, indicating partial though significant mediation by the general activation factor.

**Table 3:** Mediation Effects for All Trials > Baseline

	Effect	SE	t	p	Lower CI	Upper CI
**All Trials > Baseline, Positive Covariation with Nondecision Time (*t*0)**						
Model 1; *x* = age; *m =* First factor for 6 ROIs covarying positively with nondecision time (*t*0); *y =* nondecision time (*t*0)						
Age (*a* path)	**0.0301**	**0.0045**	**6.6472**	**0.0000**	**0.0211**	**0.0391**
General factor for 6 ROIs (*b* path)	**0.0389**	**0.0062**	**6.2707**	**0.0000**	**0.0266**	**0.0513**
Total effect for age (*c* path)	**0.0022**	**0.0003**	**7.1985**	**0.0000**	**0.0016**	**0.0028**
Direct effect for age (*c’* path)	**0.0010**	**0.0003**	**3.2527**	**0.0017**	**0.0004**	**0.0016**
Mediation effect (*ab* interaction)	**0.0012**	**0.0002**	**—**	**—**	**0.0008**	**0.0016**
**All Trials > Baseline, Negative Covariation with Nondecision Time (*t*0)**						
Model 2; *x* = age; *m =* First factor for 4 ROIs covarying negatively with nondecision time (*t*0); *y =* nondecision time (*t*0)						
Age (*a* path)	**-0.0243**	**0.0047**	**-5.1374**	**0.0000**	**-0.0337**	**-0.0149**
General factor for 4 ROIs (*b* path)	**-0.0304**	**0.0064**	**-4.7270**	**0.0000**	**-0.0432**	**-0.0176**
Total effect for age (*c* path)	**0.0022**	**0.0003**	**7.1985**	**0.0000**	**0.0016**	**0.0028**
Direct effect for age (*c’* path)	**0.0014**	**0.0003**	**4.6455**	**0.0000**	**0.0008**	**0.0021**
Mediation effect (*ab* interaction)	**0.0007**	**0.0002**	**—**	**—**	**0.0004**	**0.0012**
**All Trials > Baseline, Positive Covariation with Age**						
Model 3; *x* = age; *m =* First factor for 3 ROIs covarying positively with age; *y =* nondecision time (*t*0)						
Age (*a* path)	**0.0303**	**0.0040**	**7.4742**	**0.0000**	**0.0222**	**0.0383**
General factor for 3 ROIs (*b* path)	**0.0312**	**0.0077**	**4.0315**	**0.0001**	**0.0158**	**0.0467**
Total effect for age (*c* path)	**0.0022**	**0.0003**	**7.1985**	**0.0000**	**0.0016**	**0.0028**
Direct effect for age (*c’* path)	**0.0012**	**0.0004**	**3.4038**	**0.0011**	**0.0005**	**0.0020**
Mediation effect (*ab* interaction)	**0.0009**	**0.0003**	**—**	**—**	**0.0005**	**0.0015**

*Note. a, b, c,* = paths in mediation model as illustrated in Fig. 6, with *x* as predictor variable, *y* as outcome variable, and *m* as mediator; *a* = path from predictor to mediator; *b* = path from mediator to outcome, controlling for *a* path; *c* = total effect of predictor; *c’* = direct effect of predictor, controlling for mediator; *ab* = interaction of *a* and *b* paths representing indirect influence of *x* as mediated by *m*; effect = regression coefficient; SE = standard error; Lower/Upper CI = lower/upper bounds of 95% confidence intervals, estimated from bootstrap sampling with 10,000 samples. ROI = region of interest, derived from local maximum of significant clusters (for individual ROIs, see Table 2). Significant effects are present in bold

*Negative covariation between overall activation and nondecision time.* A similar mediation analysis was conducted for the four medial and superior frontal gyri clusters that covaried negatively with nondecision time (Table 2 and Fig. 5, Panel C). The squared multiple correlation of these four ROIs with the first factor of activation was 0.802. Mediation analyses demonstrated that the general activation factor for the negatively covarying ROIs was a significant mediator of the relation between age and nondecision time (Table 3, Model 2). As was the case with the positively covarying ROIs, the direct effect of age was reduced relative to the total effect, but remained significant, implying partial mediation.

*Positive covariation between overall activation and age*. Three ROIs in the FEF and precentral gyrus bilaterally, and the right anterior cingulate, covaried positively with age (Table 2 and Fig. 5, Panel D). The squared multiple correlation of these three ROIs with the first factor of activation was 0.755. As these three age-related ROIs were a subset of the six ROIs exhibiting the positive covariation with nondecision time, the results of the mediation analyses for the age-related ROIs yielded a pattern similar to that observed for the nondecision time-related ROIs. The general activation factor for the three age-related ROIs was a partial mediator of the age-nondecision time relation, in that the direct effect of age on nondecision time remained significant, but was reduced relative to the total effect, by including the general activation factor as a mediator (Table 3, Model 3).

*Activation for task condition contrasts.* Activation for task condition contrasts is presented in Table 4. Without age or diffusion model covariates, both the compatible and incompatible trials, compared to the neutral trials, exhibited activation in the lateral occipital cortex, bilaterally. Relative to the neutral condition, the posterior cingulate/precuneus was deactivated during the compatible trials, and the middle frontal gyrus was deactivated during the incompatible trials.

**Table 4:** Activation for Task Condition Contrasts, and Covariation with Diffusion Decision Model Parameters and Age

Cluster	Max Z	Size (voxels)	Hem	MNI Coord (mm)			BA
				*x*	*y*	*z*	
**Compatible > Neutral**							
Lateral occipital cortex	4.11	563	R	32	-84	10	19
Lateral occipital cortex	4.04	463	L	-30	-84	8	18
Parahippocampal gyrus	3.61	609	L	-26	2	-22	36
**Neutral > Compatible**							
Posterior Cingulate/Precuneus	3.93	524	R	6	-54	40	31
**Incompatible > Neutral**							
Lateral occipital cortex	4.45	697	L	-30	-88	18	19
Lateral occipital cortex	4.27	572	R	34	-80	8	19
**Neutral > Incompatible**							
Middle Frontal gyrus	4.56	731	L	-24	22	40	8
**Compatible > Neutral, Positive Correlation with Drift Rate (*v*)**							
(no significant clusters)							
**Compatible > Neutral, Negative Correlation with Drift Rate (*v*)**							
Superior parietal lobule	3.81	1242	L	-38	-52	48	40
**Compatible > Neutral, Positive Correlation with Nondecision Time (*t*0)**							
(no significant clusters)							
**Compatible > Neutral, Negative Correlation with Nondecision Time (*t*0)**							
(no significant clusters)							
**Incompatible > Neutral, Positive Correlation with Drift Rate (*v*)**							
(no significant clusters)							
**Incompatible > Neutral, Negative Correlation with Drift Rate (*v*)**							
(no significant clusters)							
**Incompatible > Neutral, Positive Correlation with Nondecision Time (*t*0)**							
Precuneus	3.87	1506	L	-4	-48	58	7
**Incompatible > Neutral, Negative Correlation with Nondecision Time (*t*0)**							
(no significant clusters)							
**Compatible > Neutral, Positive Correlation with Age**							
Frontal pole	3.87	1752	L	-22	44	-16	11
**Compatible > Neutral, Negative Correlation with Age**							
(no significant clusters)							
**Incompatible > Neutral, Positive Correlation with Age**							
Lingual gyrus	3.91	461	L	-14	-56	4	18
**Incompatible > Neutral, Negative Correlation with Age**							
(no significant clusters)							

*Note.* Max Z = highest Z values within each cluster; Hem = hemisphere; R = right; L = left; C = center; MNI = Montreal Neurological Institute; Coord = coordinates; BA = Brodmann area. Cluster regions are labeled from the Harvard-Oxford Atlas as implemented within FSL (Smith et al., 2004; http://www.fmrib.ox.ac.uk/fsl). Activation for the task condition contrasts is thresholded at Z = 2.3, Gaussian random field (GRF)-corrected at *p* < 0.05

**Fig. 7:**
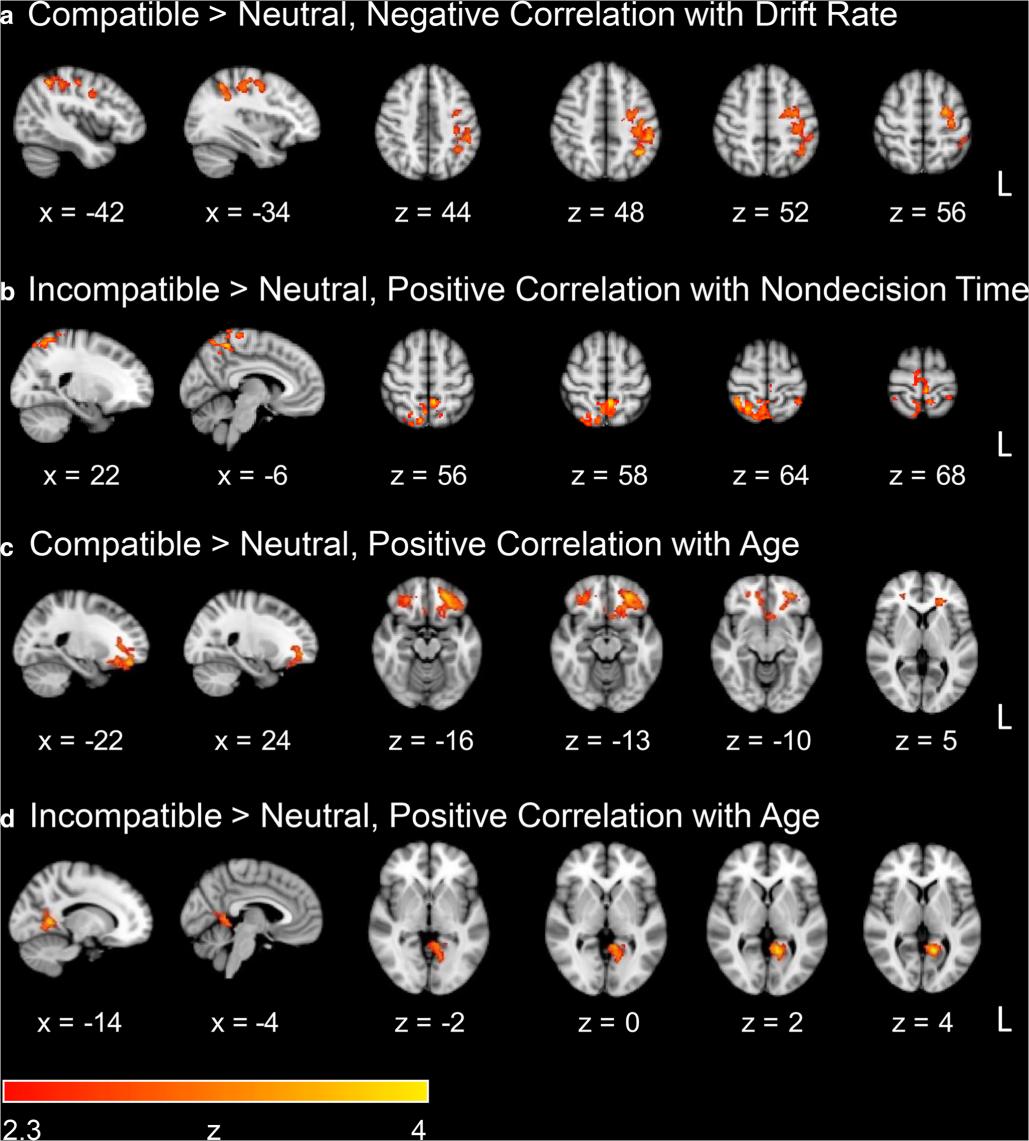
Voxelwise correlation of activation and performance for task condition effects. Panel A = negative correlation of activation and drift rate for Compatible > Neutral effect. Panel B = positive correlation of activation and nondecision time for Incompatible > Neutral effect. Panel C = positive correlation of activation and age for Compatible > Neutral effect. Panel D = positive correlation of activation and age for Incompatible > Neutral effect. Activation is thresholded at *Z* = 2.3, Gaussian random field (GRF) corrected at *p* < 0.05. Activation is overlaid on the Montreal Neurological Institute (MNI) template brain in radiological convention (left = right)

C*ovariation between compatible > neutral activation and drift rate.* Tests of drift rate and nondecision time as covariates, for the compatible > neutral activation contrast, yielded a cluster of 1,242 voxels, in the lateral parietal cortex of the left hemisphere, which exhibited a negative covariation between activation and drift rate (Table 4 and Fig. 7, Panel A). The local maximum was in the left SPL but activation extended anteriorly into the left FEF. That is, in this region, activation for compatible trials, relative to neutral trials, decreased as drift rate (the efficiency of evidence accumulation) increased. Mediation analysis indicated that the left SPL activation was not significant as a mediator of the relation between age and the increase in drift rate associated with response-compatible trials (Table 5, Model 1).

**Table 5:** Mediation Effects for Task Condition Contrasts

	Effect	*SE*	*t*	*p*	Lower CI	Upper CI
**Compatible > Neutral, Correlation with Drift Rate (*v*)**						
Model 1; *x* = age; *m =* left superior parietal lobule (SPL) cluster covarying negatively with drift rate for Compatible > Neutral contrast; *y =* Compatible > Neutral effect for drift rate (*v*)						
Age (*a* path)	-0.0001	0.0004	-0.3473	0.7293	-0.0009	0.0006
Left SPL cluster (*b* path)	**-5.4579**	**1.3687**	**-3.9876**	**0.0002**	**-8.1834**	**-2.7325**
Total effect for age (*c* path)	0.0049	0.0050	0.9812	0.3295	-0.0051	0.0149
Direct effect for age (*c’* path)	0.0042	0.0046	0.9133	0.3639	-0.0050	0.0134
Mediation effect (*ab* interaction)	0.0007	0.0022	--	--	-0.0037	0.0051
**Incompatible > Neutral, Correlation with Nondecision Time (*t*0)**						
Model 2; *x* = age; *m =* left precuneus cluster covarying positively with nondecision time (*t*0) for Incompatible > Neutral contrast; *y =* Incompatible > Neutral effect for nondecision time (*t*0)						
Age (*a* path)	0.0003	0.0005	0.6342	0.5278	-0.0006	0.0012
Left precuneus cluster (*b* path)	**0.1686**	**0.0365**	**4.6218**	**0.0000**	**0.0960**	**0.2412**
Total effect for age (*c* path)	0.0001	0.0002	0.8241	0.4124	-0.0002	0.0005
Direct effect for age (*c’* path)	0.0001	0.0002	0.5920	0.5556	-0.0002	0.0004
Mediation effect (*ab* interaction)	0.0001	0.0001	--	--	-0.0001	0.0002
**Compatible > Neutral, Positive Covariation with Age**						
Model 3; *x* = age; *m =* left frontal pole cluster covarying positively with age for Compatible >						
Neutral contrast; *y =* Compatible > Neutral effect for drift rate (*v*)						
Age (*a* path)	**0.0029**	**0.0008**	**3.6895**	**0.0004**	**0.0013**	**0.0045**
Left frontal pole cluster (*b* path)	-0.9821	0.7184	-1.3671	0.1756	-2.4126	0.4484
Total effect for age (*c* path)	0.0049	0.0050	0.9812	0.3295	-0.0051	0.0149
Direct effect for age (*c’* path)	0.0078	0.0054	1.4374	0.1547	-0.0030	0.0186
Mediation effect (*ab* interaction)	-0.0029	0.0020	--	--	-0.0078	0.0005
**Incompatible > Neutral, Positive Covariation with Age**						
Model 4; *x* = age; *m =* left lingual gyrus cluster covarying positively with age for Incompatible > Neutral contrast; *y =* Incompatible > Neutral effect for nondecision time (*t*0)						
Age (*a* path)	**0.0021**	**0.0006**	**3.5799**	**0.0006**	**0.0009**	**0.0032**
Left lingual gyrus cluster (*b* path)	-0.0328	0.0336	-0.9771	0.3316	-0.0996	0.0340
Total effect for age (*c* path)	0.0001	0.0002	0.8241	0.4124	-0.0002	0.0005
Direct effect for age (*c’* path)	0.0002	0.0002	1.1305	0.2618	-0.0002	0.0006
Mediation effect (*ab* interaction)	-0.0001	0.0001	--	--	-0.0003	0.0000

*Note. a, b, c,* = paths in mediation model as illustrated in Fig. 7, with *x* as predictor variable, *y* as outcome variable, and *m* as mediator; *a* = path from predictor to mediator; *b* = path from mediator to outcome, controlling for *a* path; *c* = total effect of predictor; *c’* = direct effect of predictor, controlling for mediator; *ab* = interaction of *a* and *b* paths representing indirect influence of *x* as mediated by *m*; effect = regression coefficient; SE = standard error; Lower/Upper CI = lower/upper bounds of 95% confidence intervals, estimated from bootstrap sampling with 10,000 samples. ROI = region of interest, derived from local maximum of significant clusters (for individual ROIs, see Table 3). SPL = superior parietal lobule. For local maxima of mediator variables, see Table 4. Significant effects are present in bold

C*ovariation between incompatible > neutral activation and nondecision time.* For the incompatible > neutral contrast, a cluster of 1,506 voxels in medial parietal cortex covaried positively with nondecision time, reflecting increased time required for encoding and response execution, as activation increased on incompatible trials relative to neutral trials (Table 4 and Fig. 7, Panel B). Although the local maximum for this cluster was in the left precuneus region, the cluster extended across the midline in the precuneus/superior parietal region. This activation was not a significant mediator of the relation between age and the increase in nondecision time associated with response-incompatible trials (Table 5, Model 2).

C*ovariation between task condition contrasts and age.* The task condition contrasts yielded two models in which activation covaried with age. First, for the compatible > neutral contrast, a cluster of 1,752 voxels, in the left frontal pole, exhibited a positive covariation between activation and age (Table 4 and Fig. 7, Panel C). Second, for the incompatible > neutral contrast, a cluster of 461 voxels, in the left lingual gyrus, activation also increased with increasing age (Table 4 and Fig. 7, Panel D). Because activation on response-compatible trials was associated with drift rate, but not nondecision time, we tested the left frontal pole activation as a mediator of the relation between age and the response-compatibility effect for drift rate. The mediating effect was not significant (Table 5, Model 3). Similarly, activation on response incompatible trials was associated selectively with nondecision time, but left lingual gyrus activation did not mediate the relation between age and the response-incompatibility effect for nondecision time (Table 5, Model 4).

## Discussion

In this experiment, we used event-related fMRI and the diffusion decision model of RT to investigate age-related differences’ response-level processing during visual feature search. We hypothesized that the age-related differences in feature search, particularly the effect of response-incompatible distractors, would be associated with nondecision time rather than drift rate, and that task-related activation would mediate the relation between age and search performance. The findings provided partial support for these hypotheses, but the mediating influence of the activation on the age-performance relation was not associated specifically with response-level processing.

### Search performance

Averaged across age, mean RT decreased on response-compatible trials, relative to neutral trials, and increased on response-incompatible trials (Fig. 3, Panel A), reflecting both positive and negative influences of the distractors on the horizontal/vertical decision to the color singleton target. Analyses with the drift diffusion model demonstrated that these response compatibility effects in mean RT were expressed differently in the decisional and nondecisional model parameters. As hypothesized, slowing for response incompatibility was evident in the nondecision time parameter *t*0 (Fig. 3, Panel C), consistent with the minimal attentional demands of feature search (Treisman, 1988, 2004). However, contrary to expectation, both compatible and incompatible distractors were associated with an *improvement* in the efficiency of evidence accumulation, as indicated by an increase in the drift rate parameter *v* (Fig. 3, Panel B). A positive relation between response-incompatible distractors and drift rate is puzzling, but it may represent a contribution to visual feature pop-out, from the differing orientations between the target and response-incompatible distractors (Fig. 1). Alternatively, the presence of task-relevant distractors, whether response-compatible or -incompatible, may facilitate response selection (as opposed to response execution), which would influence the drift rate parameter (Voss, Rothermund, Gast, & Wentura, 2013b). The diffusion decision model analyses illustrate the conflicting influences that may be hidden within the global measure of mean RT. On the response-compatible trials, the enhancement of the decisional processes related to drift rate was dominant, leading to decreased mean RT relative to neutral RT. On the response-incompatible trials, the slowing of nondecisional, encoding, and response processes was dominant, leading to the increased mean RT relative to neutral RT.

Confirming our initial hypothesis, age-related decline in search performance was associated with nondecision time rather than with drift rate (Fig. 4). This finding supports the idea that, in the context of the minimal attentional demands of feature search, the age-related differences in RT are determined largely by the encoding and response processes contributing to nondecision time (Ratcliff, 2008; Ratcliff et al., 2004). The age-related increase of approximately 2 ms per year is a small portion of the mean nondecision time, which ranged from approximately 400 ms to 800 ms, but the correlation between nondecision time and age was a large effect, with *r* > 0.60 in each of the task conditions. No age-related differences were detected for either drift rate (*v*) or boundary separation (*a*). Although age-related effects have been noted for drift rate and boundary separation, the tasks yielding age-related differences often place more complex demands on feature discrimination or memory retrieval than would feature search (Madden et al., 2009; Spaniol et al., 2006; Yang et al., 2015).

The results did not support our hypothesis that response-level processing would be a specific locus of age-related decline in feature search. We had sufficient statistical power to detect small- to medium-sized effects, but we found that the influence of response-incompatible distractors did not vary significantly with adult age. Previous research with the Stroop task (Augustinova et al., 2018; Spieler et al., 1996) and other forms of choice RT (Machado et al., 2009; Maylor & Lavie, 1998) have reported an age-related increase in vulnerability to the effect of response incompatibility, but age constancies have also been obtained (Atwi et al., 2018; Hsieh & Lin, 2014; Kramer et al., 1994; Madden & Langley, 2003). One possibility is that, in the context of the minimal attentional demands of feature search, age-related deficits in performance are more clearly evident when the task challenges the more sensory-level initial stages of feature registration than later, response-dependent stages (Madden et al., 2014; Monge & Madden, 2016).

### Search-related activation

We hypothesized that task-related activation would mediate the relation between age and search performance. As a measure of overall task-related activation, we first obtained the average activation for all three task conditions, relative to the implicit baseline. This contrast (Fig. 5, Panel A) yielded extensive activation of the frontoparietal network, which is commonly obtained in visual search and attention tasks (Corbetta et al., 2008; Katsuki & Constantinidis, 2014; Nobre & Mesulam, 2014; Wei et al., 2011). For this contrast, activation was related to nondecision time, but not to either drift rate or boundary separation. Six local maxima of this overall activation, located in the FEF bilaterally and left IPL, were correlated positively with nondecision time (Fig. 5, Panel B). The relation to nondecision time reflects the combined contributions of both relatively early, sensory-level visual feature encoding and later, motor-dependent response execution processes. The dorsal frontoparietal location of these activated regions, particularly the FEF, suggests a greater contribution from the visual encoding processes, consistent with previous findings regarding the oculomotor network and visual salience (Fecteau & Munoz, 2006; Moore & Armstrong, 2003; Thompson & Bichot, 2005; Zhou & Desimone, 2011).

Critically, the nondecision-time-related activation (the first factor of the six local maxima) was a significant mediator of the relation between age and nondecision time (Table 3, Model 1). Also, four clusters, primarily in the left medial and superior frontal gyri, covaried negatively with nondecision time (Fig. 5, Panel C), which may represent a suppression of the default network during task performance (Lustig et al., 2003). As was the case for the positively correlated regions, the common factor for the four negatively correlated regions was a significant mediator of the relation between age and nondecision time (Table 3, Model 2). Thus, supporting our initial hypothesis, the effect of age on nondecision time was indirect and due, at least in part, to a pattern of increased and decreased activation in frontoparietal regions. For both the positively and negatively correlated regions, the mediation of the age-nondecision-time relation was partial, in the sense that the direct effect of age remained significant even when the influence of the activation was taken into account.

The mediating role of activation, however, was expressed in the mean activation for the combined task conditions and did not extend to the individual task condition effects (cf. Salthouse et al., 2015). For both response-compatible and -incompatible trials, mean activation was higher in the lateral occipital cortex, bilaterally, compared to the neutral trials (Table 4), which may reflect additional visual processing of response-relevant distractors. We conducted tests of the activation-performance relation, for each of the response compatibility task conditions, by correlating activation in each condition (relative to activation on neutral trials) with drift rate and nondecision time (in each case, relative to their levels on neutral trials). On response-compatible trials, the activation and drift rate effects covaried negatively, such that increasing drift rate was associated with decreasing activation (Fig. 7, Panel A). The local maximum was in the left SPL, extending anteriorly into the left FEF. This finding is consistent with the Liu and Pleskac (2011) model of evidence accumulation, which proposes that variables leading to an increase in the rate of evidence accumulation will lead to a decrease in task-related activation, by decreasing the total amount of evidence required for a decision (see also Lustig & Buckner, 2004). Response-compatible distractors share the target feature of orientation as well as the associated response, and this activation-drift rate correlation may reflect the involvement of the visual salience map noted previously in this section of the Discussion (Fecteau & Munoz, 2006; Moore & Armstrong, 2003; Thompson & Bichot, 2005; Zhou & Desimone, 2011). However, in contrast to our initial expectation, the regional activation related to the response-compatible trials was not a mediator of the relation between age and drift rate (Table 5, Model 1), suggesting an age constancy, at the neural level, in the contribution of response-compatible information during feature search.

On the response-incompatible trials, the increase in activation relative to the neutral trials was correlated positively an increase in nondecision time relative to the neutral trials (Fig. 7, Panel B). The local maximum was located in the left precuneus, but activation extended into the superior parietal cortex bilaterally. The parietal activation is surprising, because the resolution of incompatible responses should selectively activate prefrontal regions, particularly the inferior frontal gyri (Aron, Robbins, & Poldrack, 2014; Swick, Ashley, & Turken, 2008). However, activation of the superior parietal cortex has been reported for aspects of task performance associated with the inhibition and selection of motor responses (Kolodny, Mevorach, & Shalev, 2017; Randerath, Valyear, Philip, & Frey, 2017), and it is possible that the activation reflects these latter processes related to response execution (Voss, Rothermund, et al., 2013). But, as was the case for the compatible-response activation covarying with drift rate, the incompatible-response activation covarying with nondecision time did not mediate the relation between age and nondecision time (Table 5, Model 2).

For both the overall activation (i.e., relative to the implicit baseline), and the contrasts representing the specific effects of compatible and incompatible trials, we used age as a covariate to detect regions that older adults may be recruiting to support task performance. The regions of overall activation covarying positively with age (FEF and precentral gyrus bilaterally, and the right anterior cingulate) were a subset of those covarying positively with nondecision time (Fig. 5, Panels B and D). These regions associated with age as a covariate were also similar to those covarying with nondecision time in that the activation was a significant (though partial) mediator of the relation between age and nondecision time (Table 3, Model 3). In this sense, the activation of the dorsal frontoparietal regions related to nondecision time may be a proxy for chronological age, by reflecting, at a finer scale, the slowing of central nervous system functioning.

For the task-condition contrasts, two clusters covaried significantly with age: Activation in the left frontal pole covaried positively for the compatible > neutral contrast (Table 4 and Fig. 7, Panel C), and activation in the left lingual gyrus covaried positively for the incompatible > neutral contrast (Table 4 and Fig. 7, Panel D). It is not clear why these age-related effects were lateralized to the left hemisphere. Spreng et al. (2010), in their meta-analysis of task-related activation, reported that age-related differences in prefrontal activation were right-lateralized when overall task performance was relatively worse for older adults, but were left-lateralized when task performance was relatively constant with age. Given the age-related constancy in drift rate (Fig. 4), the present findings are consistent with the Spreng et al. suggestion. However, these age-related regional activations were not mediators of the age-performance relation (Table 5, Models 3 and 4). Thus, although the age-related differences were related specifically to nondecision time, and frontoparietal activation mediated the age-nondecision-time relation, the present findings suggest that, in this highly efficient form of visual search, the age-related influence of frontoparietal activation is operative at a relatively general level, which is common to the task conditions, rather than at the response level specifically. While age-related activation was observed, this activation does not appear to be recruited to support the resolution of competing responses.

### Limitations

By including both response-compatible and -incompatible trials in this feature search task, we were able to isolate specific patterns of regional activation in relation to performance, such as the decreasing activation associated with increasing drift rate on compatible trials, and the increasing activation associated with increasing nondecision time on incompatible trials. However, the effects of compatible and incompatible trials are not exactly comparable, in that the compatible distractors share both the response-relevant feature (horizontal or vertical) and the associated response with the target, whereas the incompatible distractors differ from the target in both the response-relevant feature and the associated response. Thus, additional research is needed to identify which aspects of target-distractor similarity (Duncan & Humphreys, 1989) are contributing to the regional activation associated with drift rate and nondecision time. Similarly, the feature search task has the advantage of minimizing the attentional demands of target detection, and though the diffusion model fit the data well, the modeling is more stable when accuracy is not close to ceiling (Ratcliff et al., 2016; Voss, Nagler, et al., 2013; Voss et al., 2015).

### Conclusions

In a highly efficient visual feature search task, the most prominent form of age-related variation in performance was an increase in nondecision time, at the rate of approximately 2 ms per year, whereas the rate of evidence accumulation and degree of cautiousness were constant with age. Increasing nondecision time covaried with activation of dorsal frontoparietal regions and deactivation of medial and superior prefrontal regions. The relation of age to nondecision time was indirect, operating through the pattern of frontoparietal activation and deactivation. Response-compatible and incompatible trials were associated with specific patterns of activation in medial and superior parietal cortex, and FEF, indexed by drift rate in the case of response-compatible trials and by nondecision time in the case of response-incompatible trials. These specific effects of response-level processing, and their relation to activation, did not vary significantly with age. These findings suggest that when the attentional demands of target detection are reduced, the task-related neural activation related to age represents the nondecisional processes (e.g., visual encoding and response execution) common to the task conditions, rather than the recruitment of neural regions to support other, more specific, task demands.

#### Author Note

This research was supported by NIH research grant R01 AG039684 and R56 AG052576. We are grateful for assistance from Maria Boylan, David Hoagey, Sally Cocjin, Lauren Packard, Micah Johnson, Jesse Honig, Alex Lee, Max Horowitz, Kristin Sundy, Emily Parks, Ying-hui Chou, Michele Diaz, Angela Cook, Shivangi Jain, and Hua Huang.

## Electronic supplementary material

ESM 1 (DOCX 14 kb)

ESM 2 (DOCX 15 kb)

ESM 3 (DOCX 17 kb)
