# Supplementary material for: Response-level processing during visual feature search: Effects of frontoparietal activation and adult age
Source: Atten Percept Psychophys. 2019 Aug 2;82(1):330–49. doi: 10.3758/s13414-019-01823-3 (PMC6995405; doi:10.3758/s13414-019-01823-3)
Supplement: Supplementary file 1 — (DOCX 14 kb) [file 13414_2019_1823_MOESM1_ESM.docx]

**Supplementary Material**

*Reaction Time for Correct and Incorrect Responses*

To characterize the relation between RT and accuracy, we conducted an ANOVA of the mean RT for correct and incorrect responses for each participant in each task condition (Table S1), averaging the two responses (horizontal, vertical). The response type (correct, incorrect), and task condition (compatible, neutral, incompatible) were within-subjects variables. The main effect of response type was significant, *F*(1, 73) = 13.96 , *p* < 0.0004, η*_p_*^2^ = 0.161, representing a 68 ms faster response for errors than for correct responses (i.e., errors are fast guesses). Neither the main effect of task condition nor the Response Type x Task Condition interaction was significant. We then calculated a fast-guess difference score (correct RT – incorrect RT) for each participant in each task condition. This score was not correlated with age within any of the conditions, with *r* < 0.10 in each case. Fast guesses are typically observed when response accuracy is high but some degree of speed emphasis is present (Ratcliff & Rouder, 1998). This pattern suggests that the speed-accuracy emphasis is relatively constant across age and task condition, and that the data are suitable for the reported diffusion model analyses.

*Diffusion Decision Model for Reaction Time*

Responses were coded as correct (upper threshold) versus error (lower threshold). Trials on which participants either responded in < 300 ms (< 1% of all trials) or failed to respond (< 1% of all trials) were removed prior to analysis. The mean starting point of the decision process was fixed to be symmetric as required for diffusion model analyses on accuracy data. Drift rate (*v*) and nondecision time (*t*0) were allowed to vary across task condition (compatible, neutral, incompatible). The difference between the correct and error response thresholds (boundary separation; *a*) is assumed to remain constant across trials and thus was modeled for each participant, without relation to task condition (Ratcliff, Smith, Brown, & McKoon, 2016; Voss, Voss, & Lerche, 2015). Thus, the model comprised 10 free parameters: 3 drift rates, 3 nondecision times, separation, and inter-trial-variabilities of start point, drift, and nondecision times. Further details on model parameters are provided by Voss et al. (2013). The Kolmogorov-Smirnov criterion was used for the parameter search (Voss, Rothermund, & Voss, 2004), because this method is highly robust and efficient in the case of an intermediate number of trials (Veronika Lerche & Voss, 2017; V. Lerche, Voss, & Nagler, 2017).

Model fit was assessed with a Monte Carlo simulation. For this purpose, 1000 random data-sets were simulated with the construct-samples routine from the fast-dm package. Simulations were based on 1000 different parameter-sets that were drawn randomly from the multivariate normal distribution defined by means, variances and covariances of the estimated parameters, using the mvtnorm package for *R* (https://CRAN.R-project.org/package=MVT). The diffusion model was fit to simulated data sets to obtain a distribution of fit values for data that is based on a diffusion process. The 5% quantile of this distribution of fit values (0.696) was used as the critical value to assess the fit of real data. Fit was worse than this critical value for 5 out of 80 participants (6%). This number is very close to the expected value of 5%, and thus we view the fit of the diffusion model as being acceptable for these data.

*Correlations Among the Diffusion Decision Model Parameters*

Correlations among the diffusion model parameters are presented in Table S2. Boundary separation and drift rate were correlated negatively, representing a slower accumulation of evidence as cautiousness increased, as reported by Verdonck and Tuerlinckx (2016). Drift rate and nondecision time were independent, as were boundary separation and nondecision time. This pattern remained when the correlations were partialed for age.

**References**

Lerche, V., & Voss, A. (2017). Retest reliability of the parameters of the Ratcliff diffusion model. *Psychological Research, 81*(3), 629-652. doi: 10.1007/s00426-016-0770-5

Lerche, V., Voss, A., & Nagler, M. (2017). How many trials are required for parameter estimation in diffusion modeling? A comparison of different optimization criteria. *Behav Res Methods, 49*(2), 513-537. doi: 10.3758/s13428-016-0740-2

Ratcliff, R., & Rouder, J. N. (1998). Modeling response times for two-choice decisions. *Psychological Science, 9*(5), 347-356. doi: 10.1111/1467-9280.00067

Ratcliff, R., Smith, P. L., Brown, S. D., & McKoon, G. (2016). Diffusion decision model: Current issues and history. *Trends in Cognitive Sciences, 20*(4), 260-281. doi: 10.1016/j.tics.2016.01.007

Verdonck, S., & Tuerlinckx, F. (2016). Factoring out nondecision time in choice reaction time data: Theory and implications. *Psychol Rev, 123*(2), 208-218. doi: 10.1037/rev0000019

Voss, A., Nagler, M., & Lerche, V. (2013). Diffusion models in experimental psychology: a practical introduction. *Exp Psychol, 60*(6), 385-402. doi: 10.1027/1618-3169/a000218

Voss, A., Rothermund, K., & Voss, J. (2004). Interpreting the parameters of the diffusion model: an empirical validation. *Mem Cognit, 32*(7), 1206-1220.

Voss, A., Voss, J., & Lerche, V. (2015). Assessing cognitive processes with diffusion model analyses: a tutorial based on fast-dm-30. *Front Psychol, 6*, 336. doi: 10.3389/fpsyg.2015.00336
