# Supplementary material for: Response-level processing during visual feature search: Effects of frontoparietal activation and adult age
Source: Atten Percept Psychophys. 2019 Aug 2;82(1):330–49. doi: 10.3758/s13414-019-01823-3 (PMC6995405; doi:10.3758/s13414-019-01823-3)
Supplement: Supplementary file 2 — (DOCX 15 kb) [file 13414_2019_1823_MOESM2_ESM.docx]

Table S1

*Mean Reaction Time for Correct and Incorrect Responses by Task Condition*

Compatible Neutral Incompatible

Correct Responses 706 (95) 719 (100) 728 (101)

Incorrect Responses 677 (244) 644 (166) 626 (156)

*Note.* Values are means in ms, with SD in parentheses.
