# Supplementary material for: Response-level processing during visual feature search: Effects of frontoparietal activation and adult age
Source: Atten Percept Psychophys. 2019 Aug 2;82(1):330–49. doi: 10.3758/s13414-019-01823-3 (PMC6995405; doi:10.3758/s13414-019-01823-3)
Supplement: Supplementary file 3 — (DOCX 17 kb) [file 13414_2019_1823_MOESM3_ESM.docx]

Table S2

*Correlations Among Diffusion Decision Model Parameters for Search Performance*

*v*_incomp *v*_neut *t*0_comp *t*0_incomp *t*0_neut *a*

*v*_comp 0.531*** 0.588*** 0.132 0.061 0.021 -0.327**

*v*_incomp -- 0.579*** -0.113 -0.050 -0.165 -0.368***

*v*_neut -- -- 0.097 -0.080 0.023 -0.324**

*t*0_comp -- -- -- 0.935*** 0.930*** 0.181

*t*0_incomp -- -- -- -- 0.911*** 0.155

*t*0_neut -- -- -- -- -- 0.177

With Age Partialed

*v*_comp 0.532*** 0.593*** 0.128 0.0380 -0.0129 -0.338**

*v*_incomp -- 0.579*** -0.133 -0.053 -0.197 -0.370***

*v*_neut -- -- -0.084 -0.062 0.067 -0.320**

*t*0_comp -- -- -- 0.894*** 0.888*** 0.117

*t*0_incomp -- -- -- -- 0.857*** 0.083

*t*0_neut -- -- -- -- -- 0.113

*Note.* Values are Pearson *r*. *v*_comp = compatible trial drift rate; *v*_incomp = incompatible trial drift rate; *v*_neut = neutral trial drift rate; *t*0_comp = compatible trial nondecision time; *t*0_incomp = incompatible trial nondecision time; *t*0_neut = neutral trial nondecision time; *a* = boundary separation.

Table S2 continues

Table S2, continued

**p* < 0.05

***p* < 0.01

****p* < 0.001
